# Gene expression changes in subcutaneous adipose tissue due to Cushing's disease

**DOI:** 10.1530/JME-15-0119

**Published:** 2015-10

**Authors:** Irit Hochberg, Innocence Harvey, Quynh T Tran, Erin J Stephenson, Ariel L Barkan, Alan R Saltiel, William F Chandler, Dave Bridges

**Affiliations:** 1 Institute of Endocrinology, Diabetes and Metabolism, Rambam Health Care Campus, Haifa, Israel; 2 Life Science Institute, University of Michigan, Ann Arbor, MI, USA; 3 Physiology, UTHSC, Memphis, TN, USA; 4 Preventive Medicine, UTHSC, Memphis, TN, USA; 5 Internal Medicine, University of Michigan, Ann Arbor, TN, USA; 6 Neurosurgery, University of Michigan, Ann Arbor, MI, USA; 7 Pediatrics, UTHSC, Memphis, TN, USA

**Keywords:** Cushing's syndrome, lipolysis, insulin resistance, glucocorticoid, lipogenesis, RNA sequencing, transcriptome

## Abstract

Glucocorticoids have major effects on adipose tissue metabolism. To study tissue mRNA expression changes induced by chronic elevated endogenous glucocorticoids, we performed RNA sequencing on the subcutaneous adipose tissue from patients with Cushing's disease (*n*=5) compared to patients with nonfunctioning pituitary adenomas (*n*=11). We found a higher expression of transcripts involved in several metabolic pathways, including lipogenesis, proteolysis and glucose oxidation as well as a decreased expression of transcripts involved in inflammation and protein synthesis. To further study this in a model system, we subjected mice to dexamethasone treatment for 12 weeks and analyzed their inguinal (subcutaneous) fat pads, which led to similar findings. Additionally, mice treated with dexamethasone showed drastic decreases in lean body mass as well as increased fat mass, further supporting the human transcriptomic data. These data provide insight to transcriptional changes that may be responsible for the comorbidities associated with chronic elevations of glucocorticoids.

## Introduction

Cushing's disease, or persistently high circulating levels of cortisol secondary to a pituitary adenoma, leads to significant truncal obesity and diabetes (Cushing 1932). Obesity and diabetes are major factors in morbidity and mortality in Cushing's disease (Ntali *et al*. 2015). Cushing's disease is very rare, with an incidence of 1.2–2.4 per million (Lindholm *et al*. 2001), but iatrogenic Cushing's syndrome, caused by chronic glucocorticoid treatment, is very common and leads to similar clinical manifestations.

Numerous studies have shown that glucocorticoids have profound effects on adipose tissue metabolism, including the promotion of adipocyte differentiation (Hauner *et al*. 1987) and induction of lipolysis and lipogenesis (Divertie *et al*. 1991, Samra *et al*. 1998, Kršek *et al*. 2006, Campbell *et al*. 2011). Glucocorticoids, through binding to the glucocorticoid receptor, exert transcriptional induction and repression of numerous genes (Reddy *et al*. 2009, Surjit *et al*. 2011). Despite the widespread chronic glucocorticoid exposure, there have been no human *in vivo* studies on global gene expression changes in the adipose tissue in response to long-term exposure to glucocorticoids.

To study the effect of excess endogenous glucocorticoids on adipose tissue, we used RNA sequencing of adipose tissue biopsies from Cushing's disease patients and controls with non-secreting adenomas. We found a distinctive pattern of changes in many transcripts that are highly associated with Cushing's disease. Many of these genes explain previously observed metabolic effects of excess glucocorticoids described *in vitro*, in both animal models and humans. These include enhanced fatty acid and triglyceride biosynthesis, protein degradation, activation of glycolysis and reductions in immune responses.

## Materials and methods

### Patient recruitment

The study was approved by the institutional review board of the University of Michigan Medical System. Written informed consent was obtained from all of the patients. Patients were recruited consecutively from those undergoing a transsphenoidal adenomectomy at the University of Michigan for Cushing's disease or nonfunctioning pituitary adenoma over a 12-month period. Exclusion criteria were age <18, current hormone treatment including glucocorticoids, malignancy, inflammatory disease, diabetes type 1 and established pituitary hormone deficiencies. For each patient, a data sheet was completed including, age, sex, anthropometric measurements, diagnosis of hypertension, diabetes, results of blood tests and medications. Fasting blood samples were assayed for glucose (Siemens Advia 1800, Deerfield, IL, USA) and insulin (Life Technologies) as instructed by the manufacturers.

### Subcutaneous fat biopsy

During the course of pituitary surgery, a routine subcutaneous fat graft for sealing the surgical field is taken immediately after anesthesia but before glucocorticoid treatment. Approximately 500 mg of this fat graft was used in this study. For *ex vivo* lipolysis assay, ∼100 mg fresh adipose tissue was utilized; ∼200 mg was snap frozen in liquid nitrogen and stored at −80 °C for RNA preparation and ceramide analysis.

### Lipolysis assay

Adipose tissue pieces (25 mg) were pre-incubated for 15 min in KRBH buffer (Sigma) at 37 °C and then incubated for 1 h at 37 °C in 300 ml KRBH in duplicate. Glycerol was assayed in supernatants using a glycerol assay kit (Sigma) as instructed by the manufacturer.

### Treatment of animals with dexamethasone

Twenty-four C57BL/six adult male mice were purchased from the Jackson Laboratory (Bar Harbor, ME, USA) at 9 weeks of age. Following a 1-week acclimation period, mice were either treated with 1 mg/kg per day of dexamethasone (Sigma–Aldrich) in their drinking water (*n*=12) or used as controls (*n*=12). All animal procedures were approved by the University of Tennessee Health Science Center Institutional Animal Care and Use Committee. Animal body weight and body composition was determined weekly using an echoMRI 2100. Food was weighed weekly, with food intake determined as the decrease in food weight per mouse per week per cage. All mice were provided with access to water *ad libitum* and a standard rodent diet throughout the study. After 12 weeks of treatment, mice were fasted for 16 h and were sacrificed by cervical dislocation at ZT3 after isoflurane anesthesia. Following cervical dislocation, a sagittal incision was made along the medioventral surface of each mouse and the skin was carefully pulled back to expose the subcutaneous fat depots. The incision was extended along the anterior surface of each hind limb to allow careful dissection of the inguinal fat pads. A small incision was then made into the rectus abdominus muscle to expose the abdominal cavity. The epididymal fat pads were identified and carefully dissected out. The right fat pads from each mouse were weighed and snap frozen in liquid nitrogen for later analysis.

### Insulin tolerance test

Insulin tolerance was assessed after 12 weeks of dexamethasone treatment (21 weeks of age). Following a 6-h fast, mice were given i.p. injections of insulin (Humulin R, Lilly, Indianapolis, IN, USA) at a concentration of 1 mU/g. Blood glucose was determined at 15-min intervals post-injection using a One Touch Ultra Glucometer (Lifescan).

### Grip test

Grip strength was measured at baseline, 4, 8 and 12 weeks following treatment using a Chatillon digital force gauge (AMETEK, Berwin, PA, USA). Mice were placed on the grid with all four paws in contact with the apparatus and slowly pulled backwards by the tail. Mice were given five trials with about 10 s rests between trials. Grip strength was measured by the average peak torque (*n*) over the five trials.

### Quantitative real-time PCR

RNA was extracted with the PureLink RNA mini kit (Life Technologies). The synthesis of cDNA from 1 μg of RNA was performed using the High Capacity Reverse Transcription Kit (Life Technologies). cDNA and primers were added to Power SYBR Green PCR Master Mix (Life Technologies) in accordance with the manufacturer's guidelines and subjected to quantitative real-time PCR as previously described (Lu *et al*. 2014). The primer sequences used are listed in Table 1. mRNA expression levels of all genes were normalized to *ACTB* for adipose tissue and *GAPDH* for muscle tissue after confirming that these mRNAs are unaffected by dexamethasone treatment. Statistical tests were performed as described below based on tests of normality and homoscedasticity, then *P* values were adjusted for multiple comparisons based on the number of genes tested for each tissue across this manuscript.

### Ceramide determination

Ceramide analysis of tissue samples was performed by liquid chromatography-triple quadrupole mass spectrometry according to a modified version of the protocol reported by Kasumov *et al*. (2010). Briefly, frozen tissue samples were pulverized under liquid nitrogen, then 20 mg portions were extracted using 1.6 ml of a 2:1:0.8 mixture of chloroform:methanol:water containing internal standards (50 ng each of C17 and C25 ceramide and C12 glucosylceramide per sample). The organic layer of the extract was dried under nitrogen gas and reconstituted in 100 μl of 60:40 acetonitrile: isopropanol (Bligh & Dyer 1959). The reconstituted extract was analyzed by electrospray ionization LC–MS/MS on an Agilent (Santa Clara, CA, USA) 6410 triple quadrupole instrument operating in positive ion multiple reaction monitoring mode. The LC column used was a Waters (Milford, MA, USA) Xbridge C18, with 2.5 μm particle diameter, and column dimensions 50 mm (length)×2.1 mm (inner diameter). Mobile phase A was 5 mM ammonium acetate, adjusted to pH 9.9 with ammonium hydroxide; mobile phase B was 60:40 acetonitrile:isopropanol. The gradient consisted of a linear ramp from 50 to 100%B over 5 min, a 20-min hold at 100%B, and re-equilibration at 50%B for 10 min. The injection volume was 25 μl. Ceramides and glucosylceramides were identified by retention time and by MS/MS fragmentation parameters and were quantitated by peak area relative to the closest matching internal standard using Agilent MassHunter Quantitative Analysis software.

### Transcriptomic analysis

Total RNA was extracted from adipose tissue using the RNEasy kit (Qiagen) and its quality was verified using the Agilent 2100 Bioanalyzer (Agilent Technologies). At the University of Michigan DNA Sequencing Core, cDNA libraries from polyA mRNA were prepared using a TruSeq cDNA synthesis kit and sequenced using a HiSeq 2000 (Illumina, San Diego, CA, USA). Samples were run on two lanes of a HiSeq 2000 (Illumina) generating 8 612 682–16 469 501 single-ended 50 bp reads per sample. These were aligned to the human genome (Enembl GRCh37.74, Genbank Assembly ID GCA_000001405.14) using the TopHat version 2.0.10 (Kim *et al*. 2013), Bowtie 2 version 2.1.0 (Langmead & Salzberg 2012) and Samtools version 0.1.18. Reads were mapped to known genes using HTseq (Anders *et al*. 2015). Gene expression was analyzed using DESeq2 version 1.2.10 (Love *et al*. 2014). These subjects corresponded to the patients described in Table 2, with the exception of subjects 29 and 31 (both Cushing's disease patients), who had clinical data but no RNAseq data.

### Statistical analysis

Descriptive statistics including means and s.e.s were determined for clinical measurements. All of the statistical tests were performed using the R package (version 3.0.2 (R Core Team 2013)). Normality assumption was checked via the Shapiro–Wilk test. Wilcoxon rank sum tests were used when data were not normally distributed. Welch's *t*-test was performed if the equal variance assumption was rejected by Levene's test (car package version 2.0-19), otherwise a Student's *t*-test was used. Longitudinal measurements such as body weight, food intake, body composition and insulin tolerance tests were analyzed via mixed linear models and a *χ* ^2^ test between models with and without dexamethasone treatment as a covariate. This used the lme4 package, version 1.1-7 (Bates *et al*. 2014). Statistical significance in this study was defined as a *P*/*q*-value of <0.05. To correct for multiple hypotheses, *P* values were adjusted by the method of Benjamini & Hochberg (1995). The DESeq2 algorithm excludes genes with very high variance to improve statistical power (Love *et al*. 2014). The analysis we focused on in this manuscript was without adjustment for BMI or age and is presented in Supplementary Table 1, see section on supplementary data given at the end of this article, with gene set enrichment analyses (GSEA) in Supplementary Tables 2 and 3. A model controlled for BMI as a linear covariate or stratified into obese or non-obese subjects is presented in Supplementary Tables 4 and 5. A model controlled for both BMI and age was also constructed and is presented in Supplementary Table 6. To ensure that we did not miss any genes that had a high fold change, but that DESeq2 did not perform statistical tests for, we manually inspected genes that had an expression at >50 reads and a fold change of >2.5, but did not have a *P* value calculated (Supplementary Figure 1). These genes included *FADS1*, *FADS2*, *ELOVL6*, *SPP1*, *BMP3* and *AACS* (see Supplementary Table 1). All data are presented as mean±s.e. of the mean.

We used GSEA version 2.0.13 (Subramanian *et al*. 2005, Clark & Ma'ayan 2011) to determine whether our rank-ordered gene list for the comparison of Cushing's disease vs control patients is enriched in genes from gene ontology, Kyoto Encyclopedia of Genes and Genomes (KEGG), transcription factors or microRNA target gene sets (MSigDB version 4.0). The gene list was ranked based on *t*-statistics and the statistical significance of the enrichment score was determined by performing a 1000 phenotype permutation. Other settings for GSEA were left to the software defaults. All of the GSEA results are in Supplementary Tables 2 and 3 and summarized in Table 3. All code and raw data from this study are available through the Gene Expression Omnibus (GSE66446) and at http://bridgeslab.github.io/CushingAcromegalyStudy (Bridges & Tran 2015).

## Results

### Patient characteristics

**Figure 1:**
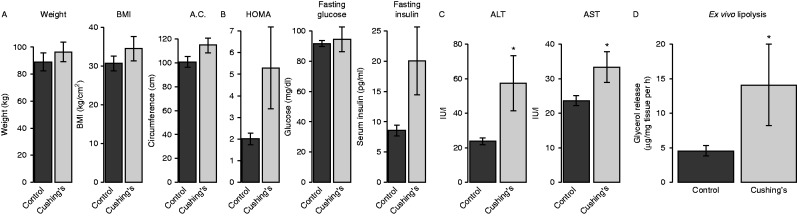
Metabolic characteristics of Cushing's disease patients in our study. (A) Morphometric data from control (non-secreting adeoma) and Cushing's disease subjects. A.C., abdominal circumference. (B) HOMA-IR score, fasting insulin and fasting blood glucose from subjects. (C) Liver enzymes from subjects (D) Glycerol release from isolated subcutaneous adipose tissue. *P<0.05.

Clinical and metabolic measurements were obtained for five Cushing's disease patients and 11 control subjects, who were admitted with non-secreting adenomas. Patient characteristics are shown in Table 2. Our Cushing's disease patients were, in general, younger and had smaller tumors than the patients with non-secreting adenomas. In the Cushing's disease cohort there was a nonsignificant elevation in body weight (*P*=0.47), BMI (*P*=0.27) and abdominal circumference (*P*=0.07, Fig. 1A), consistent with Cushing's disease patients with elevated fat mass and truncal obesity (Lamberts & Birkenhäger 1976).

We detected a nonsignificant elevation in the HOMA-IR score (2.6-fold, *P*=0.67 by Wilcoxon test, Fig. 1B), driven largely by increases in fasting insulin levels (*P*=0.30). Three out of the five Cushing's disease patients had diabetes, while only one of the 11 controls had diabetes (*P*=0.03 via *χ* ^2^ test). These data are consistent with elevated glucose intolerance in patients with Cushing's syndrome. We observed significant elevations in both alanine aminotransferase (ALT) and aspartate aminotransferase (AST) in serum from Cushing's disease patients. To evaluate lipolysis in explants from these patients, we measured glycerol release from isolated subcutaneous adipose tissue and found a 3.1-fold elevation (*P*=0.049 via Student's *t*-test). These data support previous studies that implicate elevated lipolysis (Kršek *et al*. 2006) and higher rates of nonalcoholic fatty liver disease in Cushing's disease patients (Rockall *et al*. 2003).

### Dexamethasone treatment of mice as a model of Cushing's syndrome

**Figure 2:**
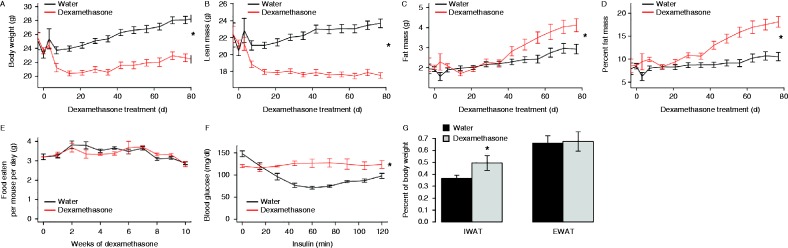
Dexamethasone treatment results in decreased lean mass and increased fat mass in mice. Weekly body weight (A), lean mass (B), fat mass (C) and percent fat (D) from control (black) and dexamethasone (red) treated mice. (E) Average food consumption per mouse per day. (F) Insulin tolerance test. Following a 6-h fast, insulin (1 mU/g) was administered via i.p. injection, and blood glucose was measured at baseline and the indicated time post injection. (G) Inguinal (IWAT) and epididymal (EWAT) fat pad weights, for right fat pads only. *P<0.05.

To validate the gene expression changes observed in human subjects, we treated C67BL/6J mice with dexamethasone, a synthetic glucocorticoid, in their drinking water to mimic the systemic effects of cortisol overproduction. These mice had an initial catabolic phase in which their body weight was rapidly reduced (Fig. 2A), an effect that was primarily due to a reduction in lean body mass (Fig. 2B). This is consistent with previously reported effects of glucocorticoids on muscle atrophy (Pleasure *et al*. 1970). After ∼5 weeks, we observed an elevation in both total fat mass and percent adiposity in the dexamethasone-treated mice (Fig. 2C and D). We did not detect any differences in food intake between the groups throughout the study (Fig. 2E). To evaluate insulin sensitivity, we performed insulin tolerance tests on these mice after 12 weeks of dexamethasone treatment and found that while they had reduced fasting glucose at this stage, they were resistant to insulin-induced reductions in blood glucose (Fig. 2E). On sacrifice after 12 weeks of dexamethasone treatment, the adipose tissue was dissected and weighed. As shown in Fig. 2F, we observed elevated subcutaneous fat mass in dexamethasone-treated animals.

### Transcriptomic analysis of human adipose tissue from Cushing's patients

**Figure 3:**
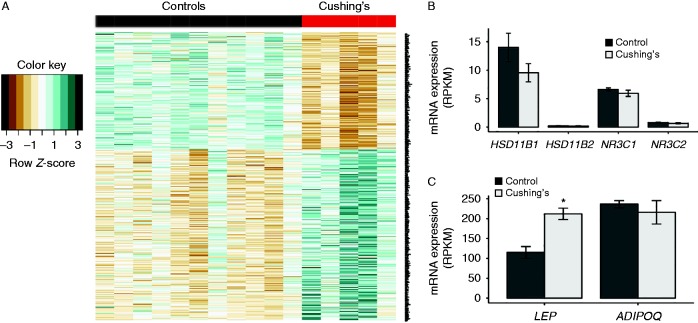
Differentially expressed transcripts in subcutaneous adipose tissue from Cushing's disease subjects. (A) Heatmap of genes with significant differential expression. The bar on the top indicates control subjects (non-secreting adenoma; black) and Cushing's subjects (red). (B) Genes involved in cortisol signaling. (C) Leptin and adiponectin mRNA levels. *q<0.05.

To determine which genes and pathways are altered in the adipose tissue in the human Cushing's disease subjects, we analyzed the transcriptome from subcutaneous adipose tissue mRNA from the five Cushing's disease patients and 11 controls. We identified 473 genes that had significantly different expressions in Cushing's disease patients; of these, 192 genes were expressed at a lower level and 281 at a higher level in the adipose tissue from the disease patients. These transcripts form a signature identifying transcriptional difference in the adipose tissue in response to long-term exposure to glucocorticoids (Fig. 3A).

To identify conserved pathways underlying these changes, GSEA was performed on these data. As summarized in Table 3, we detected the enrichment of genes in several categories involved in metabolism, including a higher expression of gene sets involved in lipid biosynthesis, glucose metabolism, activation of amino acid degradation, protein degradation and reductions in protein synthesis. We also observed reduced expression of transcripts involved in immune function. These will be discussed in subsequent sections.

We next evaluated the levels of the glucocorticoid receptor (*NR3C1*) and the mineralocorticoid receptor (*NR3C2*) and observed no significant downregulation of these receptors at the mRNA level in Cushing's patients (Fig. 3B). Another potential mechanism for negative feedback of glucocorticoid signaling is through the enzymatic activities of 11β-HSD1/2, which control the local concentrations of cortisol in adipose tissues. We observed a nonsignificant reduction in *HSD11B1* mRNA levels (24% reduced, *P* value adjusted (*P*adj)=0.49), potentially desensitizing adipose tissue to cortisol by reducing the conversion of cortisone to cortisol. The induction of leptin by glucocorticoids has been previously reported in human adipocytes (Halleux *et al*. 1998) and in human adipose tissue *in vivo* (Papaspyrou-Rao *et al*. 1997). We observed a 1.8-fold higher level of leptin (*LEP*) expression and nonsignificantly higher resistin (*RETN*) expression but no significant changes in adiponectin mRNA levels (*ADIPOQ*, *P*adj=0.94; Fig. 3C).

### Lipogenesis genes are upregulated in response to elevated glucocorticoids

**Figure 4:**
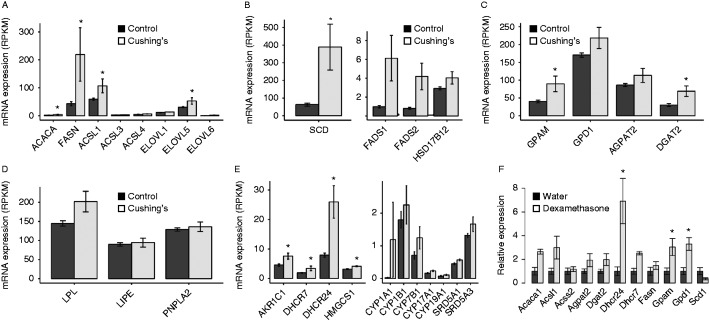
Elevated glucocorticoids result in elevated fatty acid and triglyceride synthesis genes. (A) Fatty acid synthesis genes in Cushing's disease and control patients. (B) Fatty acid desaturases in Cushing's disease patients. (C) Triglyceride synthesis genes. (D) Lipolysis genes. (E) Steroid biogenesis genes. (F) Evaluation of lipogenic genes in mouse subcutaneous adipose tissue. *q<0.05.

Increased subcutaneous fat mass is a hallmark of Cushing's syndrome and could potentially be mediated through the activation of adipogenesis or lipogenesis. Our transcriptomic data support the hypothesis that lipogenesis is activated in these tissues via the transcriptional activation of fatty acid synthesis and triglyceride synthesis. All of the major genes involved in the synthesis of fatty acids were expressed at higher levels including *ACACA*, *FASN*, *ACSL1/3/4* and *ELOVL1/5/6* (Fig. 4A). Desaturation of fatty acids is an essential aspect of *de novo* fatty acid synthesis, and we also observed elevations in all fatty acid desaturases *SCD*, *FADS1*, *FADS2* and *HSD17B12* (Fig. 4B). The triglyceride synthesis genes *GPAM*, *DGAT1*, *DGAT2*, *AGPAT2/3* and *GPD1* were also upregulated in the subcutaneous adipose tissue from Cushing's disease patients (Fig. 4C).

In spite of the increased lipid deposition and elevations of lipogenesis genes in Cushing's disease patients' adipose tissue, there have been several studies linking elevated glucocorticoids to increased lipolysis. In our patients, this was observed in *ex vivo* explants of subcutaneous adipose tissue (Fig. 1D). Among genes that may liberate fatty acids from triglycerides, lipoprotein lipase (*LPL*) was induced 1.45-fold (*P*adj=0.055) in the Cushing's disease subjects, but neither hormone sensitive lipase (*LIPE*) nor adipose triglyceride lipase (*PNPLA2*) were significantly changed at the transcriptional level (Fig. 4D). It is possible that insulin resistance due to glucocorticoids caused decreased repression of lipolysis leading to its upregulation. However, our data supports an insulin-independent activation as well, because in our explants, insulin was not present during the lipolysis assay. We detected an elevation of perilipin 4 (*PLIN4*), which is one of the proteins that coat intracellular lipid storage droplets (induced 1.45-fold, *P*adj=0.056; Supplementary Table 1).

Several genes that regulate steroid biogenesis were elevated in the adipose tissue from Cushing's disease patients as described in Fig. 4E. These include several cytochrome P450 family members, steroid reductases (*SRD5A1*, *SRD5A3*), Aldo-keto reductase family 1 member C1 (*AKR1C1*), 7-dehydrocholesterol reductase (*DHCR7*) and HMG-CoA synthase (*HMGCS1*).

To examine whether lipogenesis genes are activated in the dexamethasone-treated mice, we tested several of these genes in the subcutaneous adipose tissue from dexamethasone-treated mice and observed elevations in *FASN*, *GPAM*, *GPD1*, *ACSS2*, *ACS1*, *DGAT*, *AGPAT2*, *DHCR7/24* and *ACACA1* (Fig. 4F). In contrast to the human samples, we did not observe an elevation in the mouse isoform of *SCD* but did see instead a reduction in *Scd1* mRNA.

### Genes controlling glucose oxidation are elevated in Cushing's disease patients

**Figure 5:**
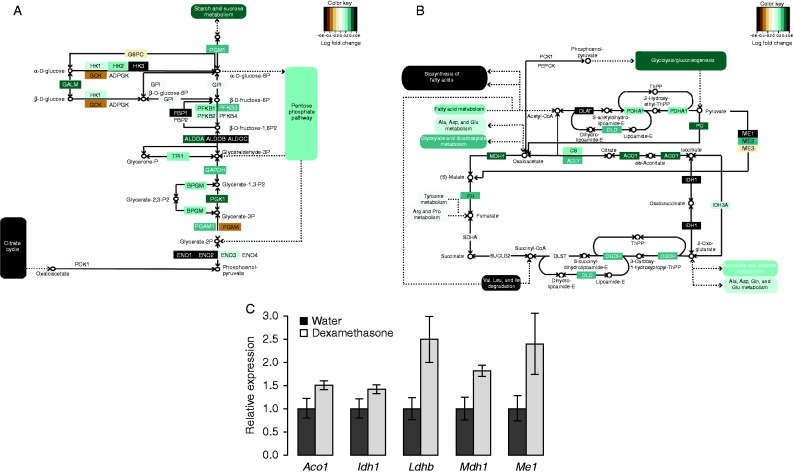
Glycolysis and glucose oxidation genes are upregulated with elevated glucocorticoids. Schematic of (A) glycolysis and (B) the TCA cycle, colored by gene expression changes in subcutaneous adipose tissue from Cushing's disease subjects. (C) qPCR analysis of selected glucose oxidation genes from mouse subcutaneous adipose tissue after 12 weeks of dexamethasone treatment. *q<0.05.

Several glucose metabolism genes, and specifically glycolysis and tricyclic acid (TCA) cycle genes were expressed at higher levels in Cushing's disease patients (Fig. 5). Strongly induced genes included *HK3*, *FBP1*, *ALDOC*, *ENO1*, *IDH1*, *ME1* and *DLAT*. Upregulations in *IDH1* and *ME1* were also noted in the mouse adipose tissue, along with other transcripts involved in glucose oxidation such as *ACO1*, *LDHB* and *MDH1* (Fig. 5C).

The major glycogen synthesis transcripts were also induced, including *GYS2*, *UGP2* and *GBE1*. This agrees with biochemical studies that implicate glucocorticoid treatment in elevated hepatic and adipose tissue glycogenesis (Engel & Scott 1951, Segal & Gonzalez Lopez 1963, Baqué *et al*. 1996). The relevance of this effect in adipose tissue has not yet been explored.

### Genes that regulate protein catabolism are upregulated in adipose tissue from glucocorticoid exposed subjects

**Figure 6:**
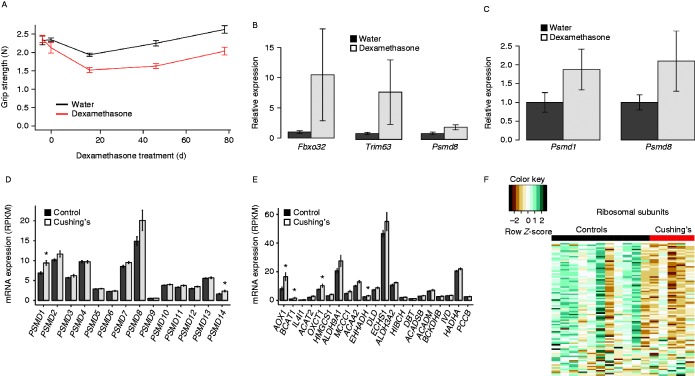
Increased glucocorticoids are associated with increased protein degradation and decreased strength. (A) Mouse grip strength (N) assessed at baseline, 4, 8 and 12 weeks of dexamethasone treatment. Muscle atrogene (B) and proteasomal transcript expression changes in gastrocnemius muscles from mice following 1 week of dexamethasone treatment. (C) Proteasomal mRNA levels from subcutaneous adipose tissue of mice treated with dexamethasone for 12 weeks. Proteasomal (D) and protein catabolism (E) transcript expression changes in subcutaneous adipose tissue from Cushing's disease and control subjects. (F) Heatmap of differentially expressed ribosomal transcripts in Cushing's disease and control subjects. * indicates *q*<0.05.

We found that two major pathways of protein homeostasis are altered in response to glucocorticoids. In concert with reductions in lean body (including muscle) mass (Fig. 2B), we observed substantial muscle weakness in mice treated with dexamethasone (Fig. 6A). In a separate cohort of mice, after 1 week of dexamethasone treatment in skeletal muscle, mRNA levels of the E3 ligases (Atrogin-1 and MuRF1) were induced as was the proteasomal gene *PSMD8* (Fig. 6B). These effects did not reach statistical significance due to variability in dexamethasone responsiveness. Similar inductions of the proteasomal genes were observed in the subcutaneous adipose tissue from the cohort of mice treated with dexamethasone for 12 weeks (Fig. 6C).

In the adipose tissue from Cushing's disease patients, we observed inductions of both the proteasomal pathways (via KEGG, net enrichment score 1.76, *P*adj=0.01; Fig. 6D) and genes involved in amino acid catabolism (Fig. 6E) and a general downregulation of ribosomal genes (Fig. 6F). Among the amino acid catabolism genes, *AOX1* (96% increase, *P*adj=0.03), *OXCT1* (40% increase, *P*adj=0.04) and *BCAT1* (80% increase, *P*=0.048) were all significantly upregulated. Together these data support the hypothesis that protein catabolism and a reduction in protein synthesis also occur in adipose tissue in response to glucocorticoid exposure.

### Genes involved in proximal insulin signaling are unchanged in adipose tissue from Cushing's disease patients

**Figure 7:**
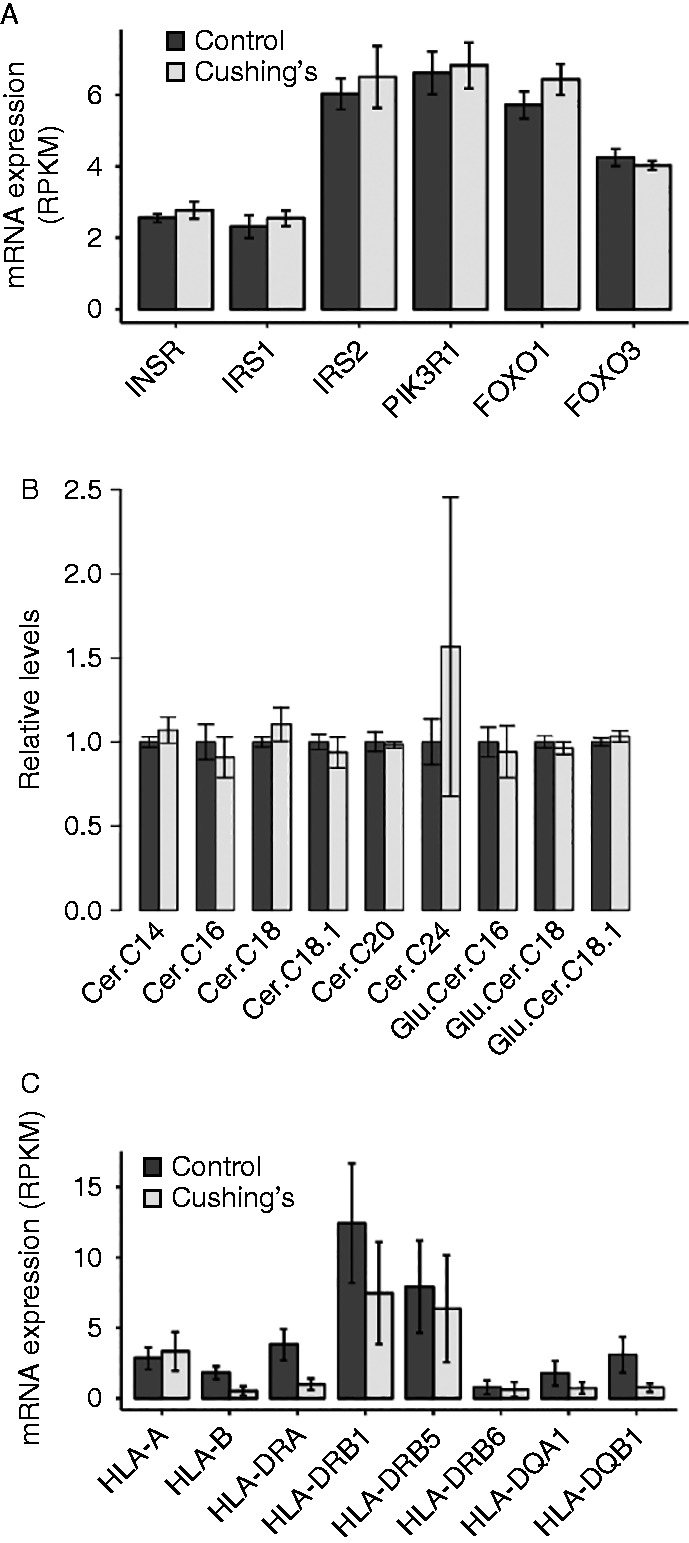
Expression of insulin signaling transcripts, ceramides and inflammatory transcripts in control vs Cushing's disease subjects. (A) Insulin signaling transcript expression levels. (B) Ceramide levels. (C) MHC complex transcript expression levels.

As described in Figs 1B and 2F, we observed insulin resistance in concert with elevated glucocorticoid levels in both mice and humans. Several mechanisms have been proposed linking glucocorticoids to insulin sensitivity including elevated lipolysis. As shown in Fig. 7A, there was a slightly higher expression of insulin pathway transcripts including *FOXO1*, the insulin receptor (*INSR*), the insulin receptor substrates *IRS1* or *IRS2* and the p85 regulatory subunit of phosphoinositide-3-kinase (*PIK3R1*), consistent with previous studies (Gathercole *et al*. 2007, Tomlinson *et al*. 2010, Hazlehurst *et al*. 2013), though in our hands none of these genes reached statistical significance. The insulin pathway was generally expressed at significantly higher levels in the Cushing's disease patients compared to controls (KEGG pathway, net enrichment score 1.84, *P*adj=0.006). These data do not support transcriptional downregulation of proximal insulin signaling genes as mediating insulin resistance in subcutaneous adipose tissue.

Changes in cell ceramide and glucosylceramide have been suggested to be important *in vitro* and in obesity- and glucocorticoid-induced insulin resistance in skeletal muscle (Adams *et al*. 2004, Aerts *et al*. 2007, Holland *et al*. 2007). To test biochemically whether ceramides may play a role in the Cushing's disease-associated insulin resistance in adipose tissue, we took a lipidomics approach to analyze the ceramide species from the adipose tissue explants of the same patients. We observed no statistically significant changes in any ceramide species (Fig. 7B, *q*>0.25).

### Inflammation

Several pathways involved in immune function were downregulated in the adipose tissue from Cushing's disease patients. This is consistent with the effects of cortisol in suppressing immune function generally. Adipose tissue leukocyte infiltration both relies on an intact immune system and responds to changes in adiposity (Lumeng & Saltiel 2011). Among immune genes, we detected reductions in several genes that form the class II major histocompatibility complex, notably *HLA-DPB2*, *HLA-DRA*, *HLA-DRB9* and *HLADQA1* (Fig. 7C). These genes normally present antigens for T-cell recruitment. Consistent with this, we observed reductions in the mRNA of *IL32*, a hormone secreted by natural killer and T lymphocytes (Dinarello & Kim 2006). We also observed a downregulation in transcripts that are interferon-gamma dependent. Together, these data support the hypothesis that the decreased T-cell activation observed with cortisol signaling also impacts adipose tissue.

### Modifying effect of obesity on glucocorticoid responsiveness

**Figure 8:**
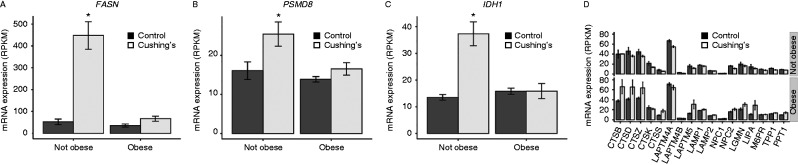
Transcript expression changes in Cushing's disease are less robust after adjusting for obesity. *FASN* (A), *PSMD8* (B), *IDH1* (C), and lysosomal (D) transcripts in non-obese and obese Cushing's subjects. * indicates *q*<0.05.

In our small cohort of Cushing's disease subjects, we examined whether some of the dramatic transcriptional changes were modified by the obesity status of the patients (based on a BMI cutoff of 30; Supplementary Tables 4 and 5). We were surprised to note that many genes that had strongly elevated transcripts in non-obese Cushing's disease patients had blunted effects in obese Cushing's disease patients. Some examples of this include *FASN*, *PSMD8* and *IDH8* (Fig. 8A, B and C). Among genes that were more strongly induced in obese patients, most of these are involved in lysosomal function, including the cathepsins (*CTSB*, *CTSD*, *CTSZ*), *LAPTM5* and *LIPA* (Fig. 8D). Although the small number of obese and non-obese Cushing's patients in our study makes these provocative observations, preliminary, it is suggestive of both a general reduction of glucocorticoid sensitivity in obese subjects and potentially an underappreciated role of lysosomes in obese patients with elevated cortisol levels.

## Discussion

In this study we have described a transcriptional signature in the adipose tissue from subjects with Cushing's disease and verified several of these changes using a mouse model of glucocorticoid treatment. We have identified several pathways that are significantly changed in response to chronic glucocorticoid exposure. Broadly, these changes reflect a shift toward more a rapid metabolism of glucose through glycolysis and the TCA cycle and a shifting of glucose and protein metabolites toward lipogenic pathways in adipose tissue. This is indicated by significant increases in glycolytic (*ALDOC*, *ENO1*, *IDH1*, *ME1*, *GALM* and *GAPDH*), proteolytic (*PSMD1/14*) and lipogenic (*ACACA*, *FASN*, *ACSL1*, *ELOVL5*, *GPAM* and *DGAT2*) transcripts in human adipose tissue, with similar transcript expression changes seen in mouse adipose and muscle tissue when treated with dexamethasone. A limitation of our human data is the difference in age between non-secreting adenoma and Cushing's disease subjects. Cushing's disease is diagnosed and treated much more rapidly, which leads to these differences. We therefore confirmed many of our human findings in a mouse model of excessive glucocorticoid treatment, wherein the mice were treated under more controlled conditions. Studies using a *Hsd11b1* knockout mouse showed similar findings to our data, including increased fat mass and decreased lean mass and strength, along with reduced insulin sensitivity (Morgan *et al*. 2014). Transcriptionally both of our studies report increases in *Dgat* mRNA, though we observed no effects of Cushing's disease on lipolytic genes (Fig. 4D) as that study did. In our study we did observe an induction of fatty acid synthesis genes in both humans and mice (Fig. 4A and F), which was not observed in the Morgan *et al*. study. Three differences could potentially explain these discrepancies. One is that in our case, dexamethasone is already active and cannot be further activated by 11β-HSD1, whereas in their study corticosterone can be both inactivated by 11β-HSD2 and reactivated by 11β-HSD1. Another key difference is the duration of treatment, which for our study was 3 months and for the Morgan *et al*. study was just over 1 month. Finally, they determined mRNA levels from gonadal adipose tissue, not subcutaneous adipose tissue, as we did in our work.

Cushing's disease patients have a significant change in fat distribution (Mayo-Smith *et al*. 1989) and higher lipogenesis, as measured by the conversion of glucose to neutral lipid *ex vivo* in the subcutaneous adipose tissue from Cushing's disease patients compared to obese controls (Galton & Wilson 1972). Higher triglyceride synthesis has also been found in animal models of Cushing's disease, including corticotropin-releasing hormone (CRH)-overproducing mice, which also have elevated glucocorticoid levels (Harris *et al*. 2013) and in dexamethasone-treated mice (Roohk *et al*. 2013). These findings are consistent with our observed elevations of lipogenesic mRNA transcripts in human and mouse subcutaneous adipose tissue. Key transcripts in this category found to be significantly upregulated include acetyl-CoA carboxylase alpha (*ACACA*), responsible for the first step of lipogenesis (the irreversible conversion of acetyl-CoA to malonyl-CoA), and glycerol-3-phosphate acyltransferase (*GPAM*), responsible for the first step in the synthesis of glycerolipids. In addition to a shift toward lipid storage, we also observed an elevated expression of glycogen synthesis mRNA transcripts in the Cushing's disease patients. Most notably of these are the significantly elevated mRNA transcripts glycogen synthase 2 (*GYS2*) and UDP-glucose pyrophosphorylase 2 (*UGP2*), both of which are required for glycogen synthesis.

Muscle wasting is a well recognized adverse event of excess glucocorticoids caused by both increased muscle proteolysis and decreased protein synthesis (Deng *et al*. 2004, Menconi *et al*. 2007). Exposure of rats to glucocorticoids activates the muscle ubiquitin-proteasome system (Wing & Goldberg 1993, Price *et al*. 1994), increasing muscle expression of proteases (cathepsins B and D, calpain) and components of the ubiquitin-proteasome pathway (Dardevet *et al*. 1995) along with the inhibition of muscle protein synthesis (Long *et al*. 2001). A study in healthy humans also found that prednisone (a synthetic corticosteroid) increases leucine oxidation, supporting our observation of elevated amino acid catabolic genes (Beaufrere *et al*. 1989). We found a higher expression of both proteasomal and amino acid degradation pathways in the adipose tissue, suggesting that a similar induction occurs in adipose tissue from our Cushing's disease subjects. We also observed elevations in lysosomal genes, though these changes appear to be restricted to obese Cushing's disease patients. The metabolic relevance of activated proteolysis in adipose tissue has not been widely explored and warrants further study.

There are several limitations to our evaluation of insulin sensitivity in this study. One aspect is that two of the three patients with Cushing's syndrome and diabetes were treated with antidiabetic medications. Secondly, it is possible that insulin resistance in these subjects/mice is mainly due to muscle or liver insulin resistance and that adipose tissue may respond to insulin in a relatively normal fashion. Glucocorticoid-induced insulin resistance is thought to be mostly secondary to the increase in free fatty acids caused by the induction of lipolysis (Geer *et al*. 2014). Results from a recent study imply that glucocorticoids do not induce insulin resistance in subcutaneous adipose tissue *in vivo* in healthy subjects (Hazlehurst *et al*. 2013), suggesting that peripheral insulin resistance may not occur in adipocytes and that whole-body insulin resistance may primarily occur in muscle and liver tissues. This is consistent with our observations of a lack of changes in proximal insulin signaling transcripts (Fig. 7A) or ceramides in our subcutaneous adipose tissue lysates (Fig. 7B).

Another limitation in our study is the small sample size, especially the number of biological replicates in the Cushing's group (*n*=5). Adding a covariate such as BMI or age in the model further reduces the sample size to two or three replicates. Although this sample size is small, it is reasonable for a rare disease such as Cushing's. Realizing our limitation, we chose DESeq2 as the statistical method for our RNAseq data. DESeq2 overcomes the small sample size problem by pooling information across genes. The maximum likelihood estimation is applied to estimate the dispersion or variance of a gene across all replicates in a group. An empirical Bayes approach is then used to get maximum *a posterior* as the final dispersion estimate. This method utilizes the available data to the maximum extent and, therefore, helps avoid potential false positives (Love *et al*. 2014).

These data provide a variety of novel transcriptional changes that may be causative of the comorbidities associated with Cushing's disease. Further studies in animals and cells using knockout or overexpression of specific transcripts may verify which of the changes is crucial in the metabolic effects of glucocorticoid effects in adipose tissue.

## Supplementary data

This is linked to the online version of the paper at http://dx.doi.org/10.1530/JME-15-0119.

## Author contribution statement

I Ho conceived the study, and D B, A R S and I Ho provided funding. W F C and A L B recruited the patients and obtained clinical data. W F C supplied the biopsies and serum samples. I Ho assayed the tissues for lipolysis and performed the serum measurements. Q T, D B, I Ha and I Ho analyzed the RNAseq data. I Ha generated the mouse data with assistance from E J S. This was analyzed by I Ha, D B and Q T. I Ho and D B wrote the manuscript.

## Figures and Tables

**Table 1:** Primer sequences used for qPCR analyses

**Gene**	**Forward sequence**	**Reverse sequence**
*Acaca*	GCTAAACCAGCACTCCCGAT	GTATCTGAGCTGACGGAGGC
*Aco1*	AACACCAGCAATCCATCCGT	GGTGACCACTCCACTTCCAG
*Acsl1*	GCCTCACTGCCCTTTTCTGA	GCAGAATTCATCTGTGCCATCC
*Acss2*	CGTTCTGTGGAGGAGCCAC	GGCATGCGGTTTTCCAGTAA
*Actb*	ATGTGGATCAGCAAGCAGGA	AAGGGTGTAAAACGCAGCTCA
*Agpat2*	CGTGTATGGCCTTCGCTTTG	TCCATGAGACCCATCATGTCC
*Dgat2*	AACACGCCCAAGAAAGGTGG	GTAGTCTCGGAAGTAGCGCC
*Dhcr7*	ATGGCTTCGAAATCCCAGCA	GAACCAGTCCACTTCCCAGG
*Dhcr24*	AGCTCCAGGACATCATCCCT	TACAGCTTGCGTAGCGTCTC
*Fasn*	GGAGGTGGTGATAGCCGGTAT	TGGGTAATCCATAGAGCCCAG
*Fbxo32*	CTTCTCGACTGCCATCCTGG	GTTCTTTTGGGCGATGCCAC
*Gapdh*	CACTTGAAGGGTGGAGCCAA	ACCCATCACAAACATGGGGG
*Gpam*	AGCAAGTCCTGCGCTATCAT	CTCGTGTGGGTGATTGTGAC
*Gpd1*	GTGAGACGACCATCGGCTG	TTGGGTGTCTGCATCAGGT
*Idh1*	CTCAGAGCTCTCTTGGACCGA	CATCTCCTTGCATCTCCACCA
*Ldhb*	AAAGGCTACACCAACTGGGC	GCCGTACATTCCCTTCACCA
*Mdh1*	GGAACCCCAGAGGGAGAGTT	TGGGGAGGCCTTCAACAAAC
*Me1*	GGACCCGCATCTCAACAAG	TCGAAGTCAGAGTTCAGTCGTT
*Psmd1*	TGCCAATCATGGTGGTGACA	ACACATCCTGACGTGCAGTT
*Psmd8*	ACGAGTGGAACCGGAAGAAC	CCGTGGTTGGCAGGAAATTG
*Rplp0*	GAAACTGCTGCCTCACATCCG	GCTGGCACAGTGACCTCACACG
*Rplp13a*	GCGGATGAATACCAACCCCT	CCTGGCCTCTCTTGGTCTTG
*Scd1*	CACTCGCCTACACCAACGG	GAACTGGAGATCTCTTGGAGCA
*Trim63*	GAGGGCCATTGACTTTGGGA	TTTACCCTCTGTGGTCACGC

**Table 2:** Clinical characteristics of Cushing's disease and control patients. Data represents mean±s.e.

**Variable**	**Cushing's disease (*n*=5)**	**Controls (*n*=11)**	***P* value**
Height (cm)	166±4.3	169±2.4	0.47
Weight (kg)	91±9.1	89±6.7	0.89
BMI	33±3.8	30±1.8	0.52
Abdominal circumference (cm)	112.4±6.4	100.65±4.4	0.16
Tumor size (cm)	0.95±0.3	1.96±0.14	0.01
Age (years)	39.8±4.5	63.4±2.7	0.0003

**Table 3:** Summarized gene set enrichment analysis of pathways. Selected pathway enriched in subcutaneous adipose tissue from Cushing's disease patients via GSEA analysis. For a complete list, see Supplementary Tables 2 and 3

**Pathway**	**Dataset**	**NES**
M_PHASE_OF_MITOTIC_CELL_CYCLE	Gene ontology	2.60*
KEGG_CITRATE_CYCLE_TCA_CYCLE	KEGG	2.41*
KEGG_BIOSYNTHESIS_OF_UNSATURATED_FATTY_ACIDS	KEGG	2.41*
REACTOME_TRIGLYCERIDE_BIOSYNTHESIS	Reactome	2.24*
PYRUVATE_METABOLISM	Gene ontology	2.24*
KEGG_VALINE_LEUCONE_AND_ISOLEUCINE_DEGRADATION	KEGG	2.16*
STEROID_BIOSYNTHETIC_PROCESS	Gene ontology	2.11*
KEGG_STARCH_AND_SUCROSE_METABOLISM	KEGG	2.08*
PROTEASOME_COMPLEX	Gene ontology	1.78*
KEGG_ALLOGRAFT_REJECTION	KEGG	−1.87*
KEGG_BASAL_CELL_CARCINOMA	KEGG	−1.86*
KEGG_RIBOSOME	KEGG	−2.33*

NES, net enrichment score; *q<0.25.
